# Insights into the water status in hydrous minerals using terahertz time-domain spectroscopy

**DOI:** 10.1038/s41598-019-45739-2

**Published:** 2019-06-25

**Authors:** Yuanyuan Ma, Haochong Huang, Sibo Hao, Kunfeng Qiu, Hua Gao, Lu Gao, Weichong Tang, Zili Zhang, Zhiyuan Zheng

**Affiliations:** 1 School of Science, China University of Geosciences, Beijing, 100083 China; 2 School of Earth Science and Resources, China University of Geosciences, Beijing, 100083 China

**Keywords:** Physics, Optics and photonics, Physics, Optics and photonics, Optics and photonics

## Abstract

The determinations of water status incorporated in hydrous minerals are of considerable significances in geoscience fields. Coincidentally, the aqueous sensitivity of terahertz radiation has motivated numerous explorations in several cross-domain applications. Terahertz time-domain spectroscopy is employed as a major probing technique coupling of traditional detecting methods to uncover the mask of water status in copper sulfate pentahydrate as well as mineral quartz in this article. Based on the quantitative identification of water status in copper sulfate pentahydrate, the water incorporated in mineral quartz is verified qualitatively. Notable differences of optical constants originating from the water content are obtained for copper sulfate pentahydrate and mineral quartz. These present works indicate that terahertz technology can be considered as a promising method to satisfy the ever-increasing requirements in hydrous mineral analyses.

## Introduction

Water is well-known for one interesting feature that the ability to organize itself systematically into other substances, such as minerals. There are approximate 500 hydrous minerals spreading all over the world^1^. It is acknowledged that water content and status are variable in hydrous minerals. During the evolution of geological mineral formations, the amount of water content in minerals has happened to change due to the long-term natural operation. In particular, the ambient temperature will significantly influence the water status and content. According to the relationship between water and the crystal structure of its host minerals, water mainly exists in three states, free hydrogen-bonded water, crystal water and structural water. Generally, free hydrogen-bonded water named adsorbed-water dehydrates at a temperature of about 120 °C. Crystal water with a specific status in the minerals’ crystal structure possesses a dehydration temperature of about 200 to 500 °C. Structural water, which presents in the form of hydroxyl, has a dehydration temperature of about 600 to 1000 °C or even higher^2^. The switch of water content can possess disproportionate impacts on the physicochemical properties of host minerals. Recently, a growing number of studies have pointed to the effects of the water content on enhancing the electrical conductivity^3^, controlling the diffusion rate of ionic species^4^, and influencing the migration and thermal stability of host minerals^5^. The variances of minerals’ properties directly reflect the evolution of the local geological environment. Thus, the quantitative and qualitative investigations of water content in hydrous minerals play a key role in mineral analyses.

**Figure 1:**
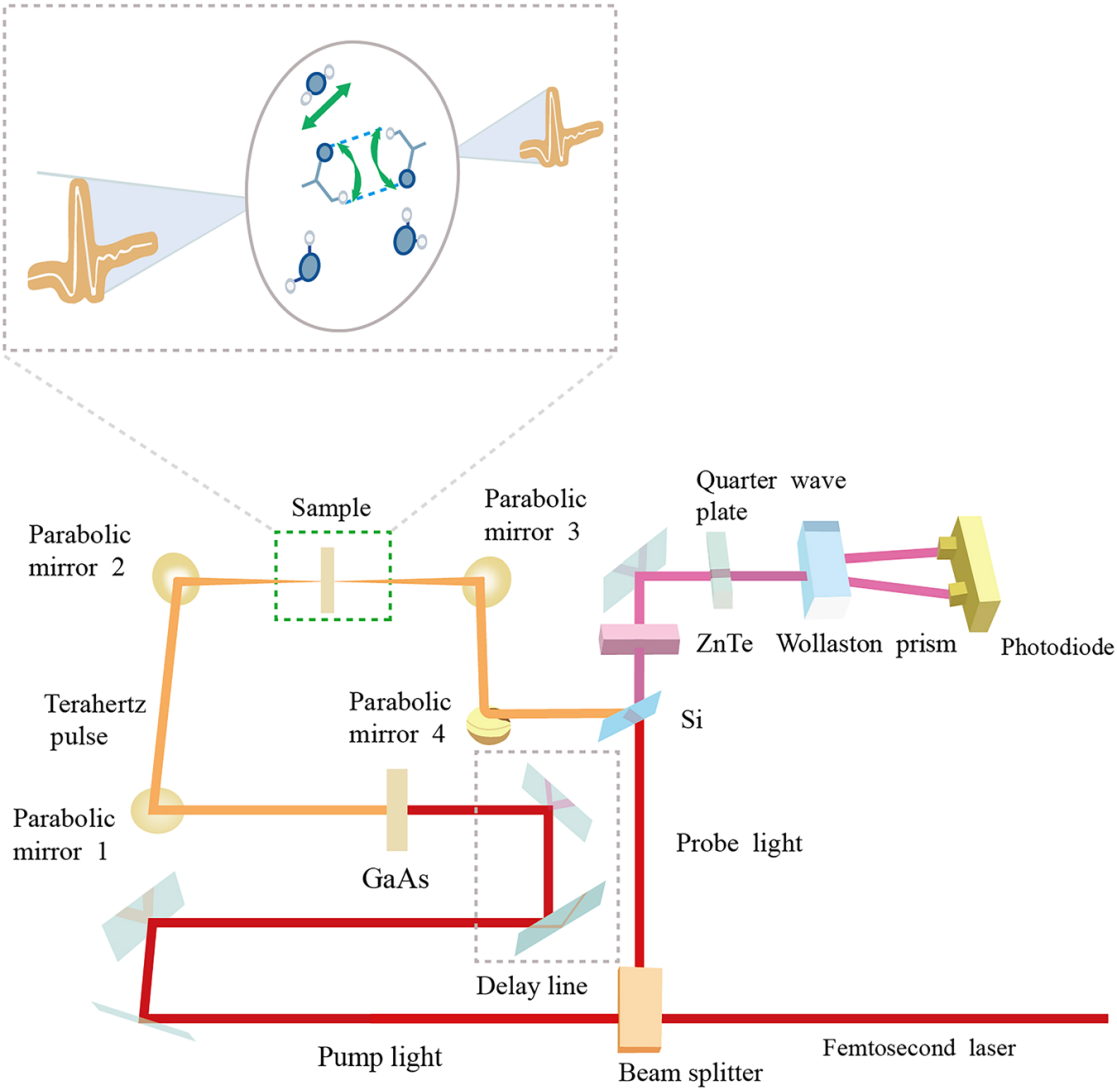
Diagram of THz-TDS experimental set-up. The enlarged part shows the schematic illustration of the vibration modes of water molecules in hydrous minerals probed by terahertz wave. Incident terahertz wave (left) and transmitted terahertz wave (right) are indicated with orange lines. Blue and white balls indicate O and H atoms, respectively.

A substantial of approaches to determining the water content of hydrous minerals are spurred by advances in measuring apparatus, such as infrared spectroscopy^6^; thermogravimetric analysis (TGA)^7^ and powder X-ray diffraction (PXRD)^8^. Though the above methods have many merits, the detection of water still requires a technique with higher sensitivity and dependency. One reason is that the vibration modes of water molecules consist of low-frequency modes (LFM) and high-frequency modes (HFM)^9^. LFM includes flexing of the water molecule (bidirectional arrow in the enlarged part of Fig. 1) and intermolecular vibrations (curving arrow in the enlarged part of Fig. 1) while HFM refers to the vibration of hydroxyl. The strong HFM absorption can obscure the weak LFM absorption during water detection. The other reason is that the water content bound as hydroxyl is in the order of parts per million (ppm), especially for nominally anhydrous minerals (NAMs). It is not always feasible to be detected by conventional methods. The exploitation of a novel technique allowing to circumvent many of the challenges thus becomes highly crucial for the fundamental understanding of water content in minerals. Terahertz time-domain spectroscopy (THz-TDS), as a state-of-the-art spectra technique over the past decade, has prevailed in biology^10–14^; oils^15,16^; and medical^17–19^ because it can provide sufficient optical information of molecular level interactions by obtaining amplitude and phase data simultaneously^20,21^. THz-TDS adopts the terahertz-wave detection with higher signal-to-noise ratio (SNR) locating in frequencies (0.1–10 THz) between the far infrared and microwave^22,23^. Terahertz wave is of lower energy^24,25^, and can be absorbed by the LFM of water molecules^26–29^. Moreover, water molecules’ dynamics driven by diverse types of thermally excited intermolecular motions are so strong that the network of hydrogen bonds (blue dashed lines in the enlarged part of Fig. 1) rearranges on sub-picosecond (sub-ps) time scales, which is in agreement with the bandwidth of terahertz wave. Consequently, THz-TDS can be deemed as an ideal tool for sensitive water determinations of hydrous minerals.

Here, we perform both the quantitative assignments of water molecules in copper sulfate pentahydrate (CuSO_4_·5H_2_O) and the characterizations of trace amounts of hydrous components that occur in mineral quartz using THz-TDS as well as other conventional analytical methods. On the one hand, it reports the terahertz frequency-dependent absorption coefficients and refractive indices of CuSO_4_·5H_2_O at various temperatures, while the water content of CuSO_4_·5H_2_O is quantitatively identified. These important optical constants manifest high correlations with the number of crystal water molecules. On the other hand, it qualitatively characterizes the water content of mineral quartz by enabling the combinations of THz-TDS with PXRD and Fourier transform infrared (FTIR) spectroscopy. The results encouragingly show that the temperature-dependent water content of mineral quartz can be characterized at terahertz frequencies. It is discovered that the dehydration process in mineral quartz presents the dependence of frequencies. These works reveal that THz-TDS will act as a potent candidate for quantitatively and qualitatively analyzing the water content and status.

## Results and Discussions

### Water determinations of CuSO_4_·5H_2_O

**Figure 2:**
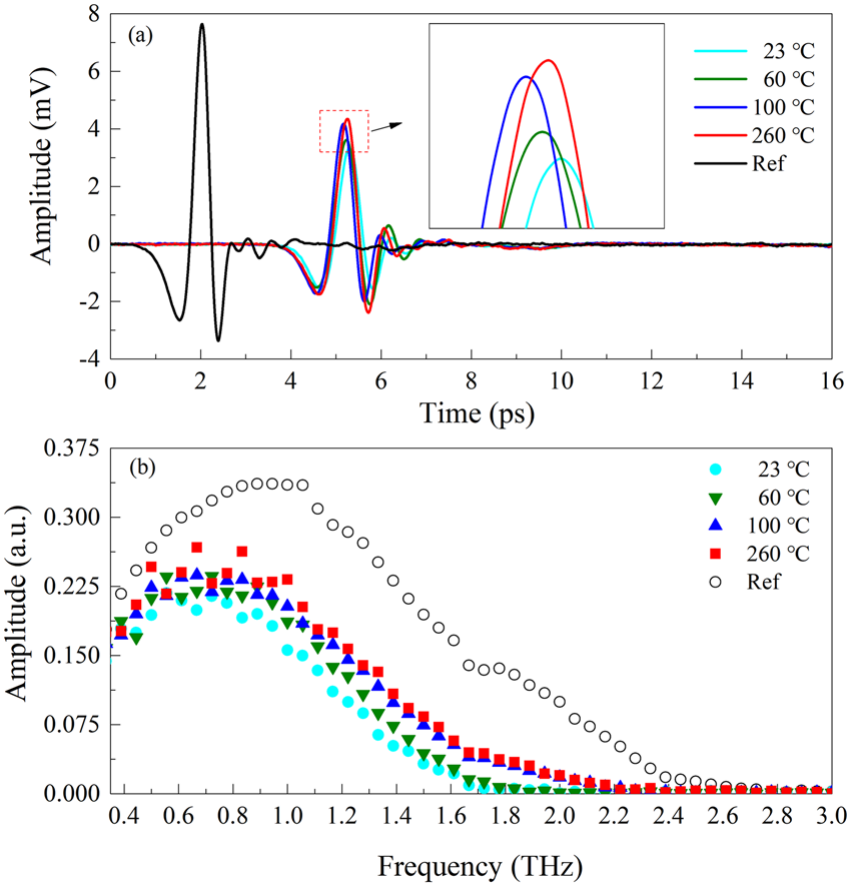
Diagram of THz-TDS (**a**) and terahertz frequency-domain spectroscopy (THz-FDS) (**b**) of CuSO_4_·5H_2_O heated to different temperatures. The inset shows the enlarged part of the plot detail.

The THz-TDS results of CuSO_4_·5H_2_O heated from 23 to 260 °C are presented in Fig. 2a. In contrast to the reference signal, the signals of four selected temperatures are time delayed and amplitudes are decayed due to the sample refraction and absorption. Almost the appearance of no phase shift under four temperatures ensures that THz-TDS is not to be distorted by the thickness of the sample. The amplitude variances of THz-TDS at different temperatures are clearly observed in the inset. Upon increasing the temperature from 23 to 260 °C, the amplitudes are seen to decrease. As a matter of fact, the amplitude of the transmission terahertz time-domain signal is proportional to the water content. In CuSO_4_·5H_2_O, crystal water molecules either coordinate with copper ions or form hydrogen bonds^30^. The variances of terahertz absorptions are mainly caused by two contributions. One is that the increase in temperatures brings about the decomposition in crystal water. Another is that the status of hydrates of copper sulfate pentahydrate has various crystal symmetry and coordination. The variances of dielectric properties will affect the optical responses to the terahertz wave^31^. Moreover, it is noted that the amplitude of the temporal signal of 260 °C attenuates significantly compared to the reference owing to the intrinsic absorption of anhydrous copper sulfate.

Figure 2b shows the Fourier transforms of Fig. 2a in the time range from 0 to 16 ps. The corresponding amplitudes of THz-FDS augment as temperatures increase. The spectral range of the reference is shown up to about 3.0 THz while it narrows down to about 2.4 THz for 100 and 260 °C as well as 2.0 THz for 23 and 60 °C owing to the different substantial absorption of the samples. The spectral range of each hydrate extends to a little higher frequency than before upon the dehydration.

**Figure 3:**
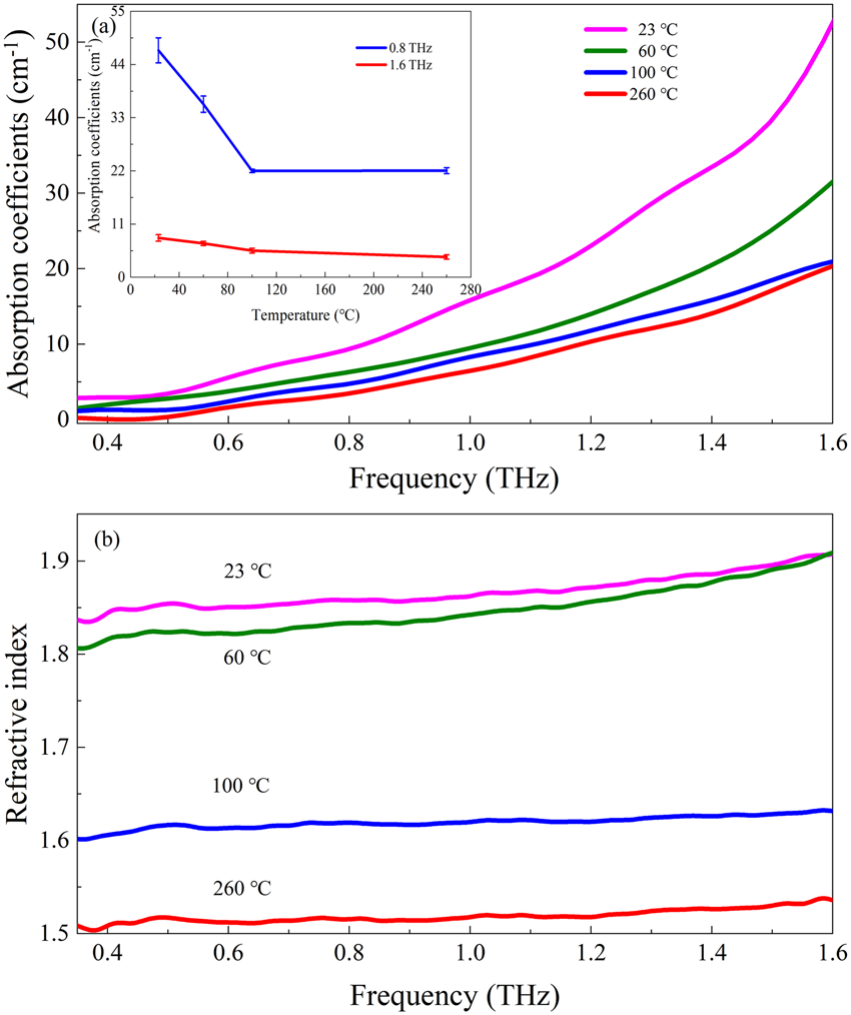
Frequency-dependent absorption coefficients (**a**) and refractive index (**b**) of CuSO_4_·5H_2_O heated to different temperatures. The inset shows the temperature-dependent absorption coefficients at 0.8 and 1.6 THz, respectively.

Figure 3 shows the frequency-dependent absorption coefficients and refractive indices of CuSO_4_·5H_2_O in the temperature range from 23 to 260 °C. One can see the same trend as the temporal signals for both the absorption coefficients and refractive indices. They all decline upon the dehydration of crystal water molecules. Figure 3a shows that the absorption coefficients of four temperatures increase rapidly, especially the temperature of 23 and 60 °C at high frequencies. The value variances of absorption coefficients at 0.8 THz and 1.6 THz are recognized in the inset. The nonlinear slopes of the two curves denote that the rate of the dehydration process is various. It is observed that with the ambient temperature increasing from 23 to 100 °C, absorption coefficients at 0.8 THz vary from 8.1 to 5.5 cm^−1^. At 1.6 THz, absorption coefficients descend from 46.9 to 22.0 cm^−1^. This phenomenon illustrates that the water molecules are more sensitive to the higher terahertz frequency. They require higher energy to overcome the hydrogen bond binding, and start to appear as the mode of intermolecular vibrations under the driving of the stronger terahertz radiation. Theoretically, the number of crystal water molecules of copper sulfate pentahydrate can be regarded as the crystal water content under the macro case at different temperatures. During the heating process, the number of dehydrated water molecules follows the order of 2, 2 and 1. The absorption coefficients and the crystallization water content are in accordance with Lambert Beer’s law. In experiments, CuSO_4_·5H_2_O transforms into copper sulfate trihydrate (CuSO_4_·3H_2_O) firstly upon heated from 23 to 60 °C. Another two water molecules are lost to form copper sulfate hydrate (CuSO_4_·H_2_O) when heated to about 100 °C. The CuSO_4_·H_2_O is converted to the copper sulfate (CuSO_4_) till the temperature increases to about 260 °C. Those experimental absorption coefficients agree well with that of above discussed theory (Supplementary Materials). And monotonous temperature dependence of the refractive indices is observed in Fig. 3b. The lower refractive index occurs at the temperature of 260 °C. The curves’ shapes continue to have a similar trend over all frequency bands.

The identical temperature-dependent trend between refractive index and absorption coefficients provides solid evidence that the two optical parameters arise from same mechanisms in CuSO_4_·5H_2_O. In summary, THz-TDS has different feedback on four kinds of hydrates of CuSO_4_·5H_2_O. The strong temperature-dependent absorption coefficients and refractive indices at terahertz frequencies can be used as enhanced quantitative clarifications of CuSO_4_·5H_2_O and its other three hydrate forms.

**Figure 4:**
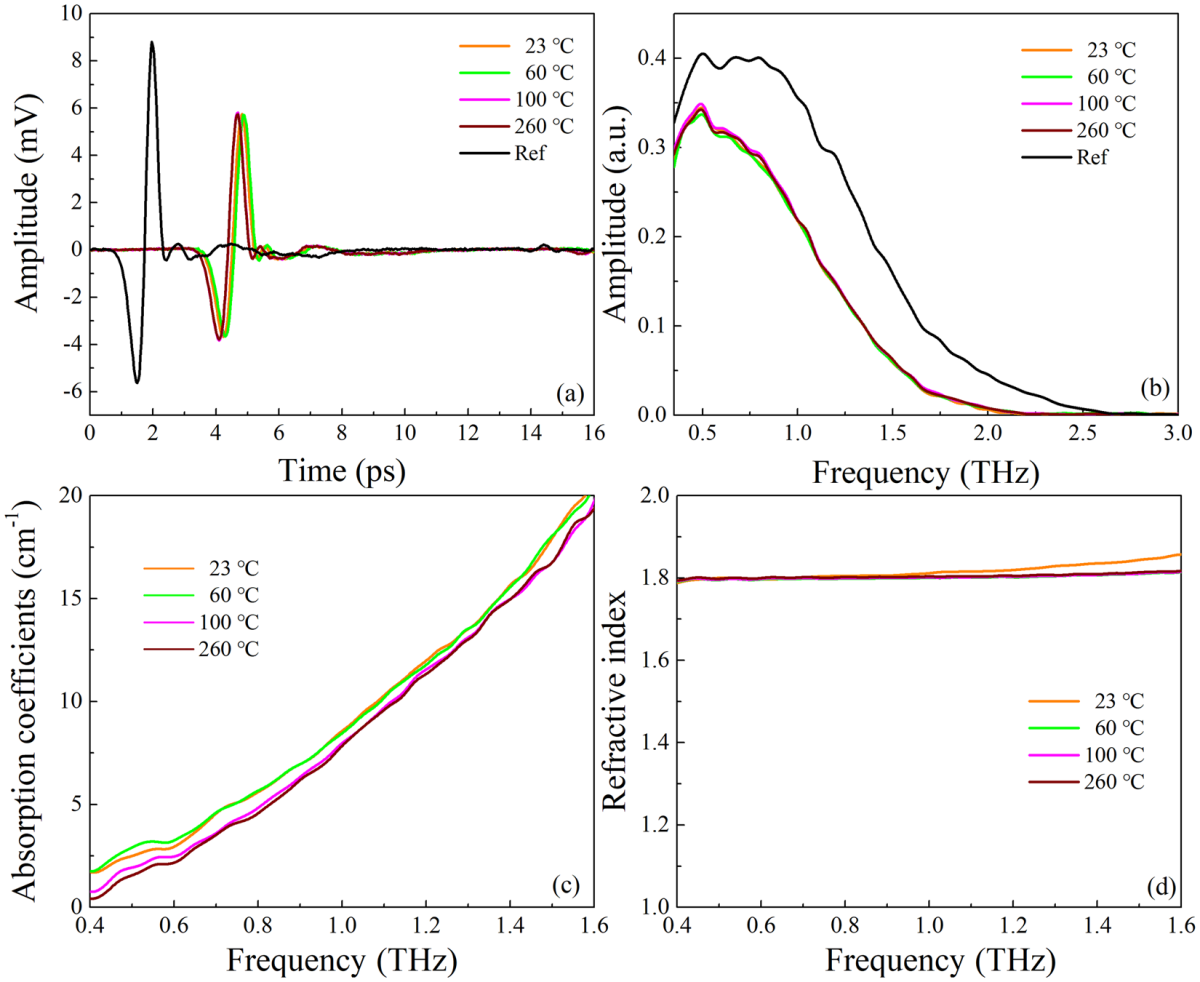
Terahertz time-domain spectra (**a**); frequency-domain spectra (**b**); absorption coefficients (**c**) and refractive index (**d**) of anhydrous CuSO_4_ after heating treatments (from 23 to 260 °C) and then cooling to room temperature.

Additionally, in order to exclude the effect of dielectric properties on terahertz absorptions, anhydrous copper sulfate is measured at different temperatures as shown in Fig. 4. It can be seen from Fig. 4(a) that the time-domain spectra of four temperatures are basically the same. Almost no distinction is observed in frequency-domain spectra (b); absorption coefficients (c) and refractive index (d). Those results prove that the dielectric properties of anhydrous copper sulfate do not vary in the temperature range from 23 to 260 °C.

### Characterizations of water in mineral quartz

Based on the above discussions of CuSO_4_·5H_2_O, it can be concluded that other similar minerals of which the chemical formulas contain a certain number of water molecules are likely to be characterized using THz-TDS. In this section, the mineral quartz will be determined by THz-TDS combining PXRD and FTIR to explore and analyze hydrous components. Mineral quartz is an earth-abundant mineral form of tetrahedrally bonded silica, and is one kind of NAMs first studied for the influence of water on its mechanical strength^32^. Compared to the crystal quartz, mineral quartz presents higher absorption coefficients at terahertz bands. (Supplementary Materials) The characterizations of water content in mineral quartz would pave the way for subsequent researches on other NAMs.

**Figure 5:**
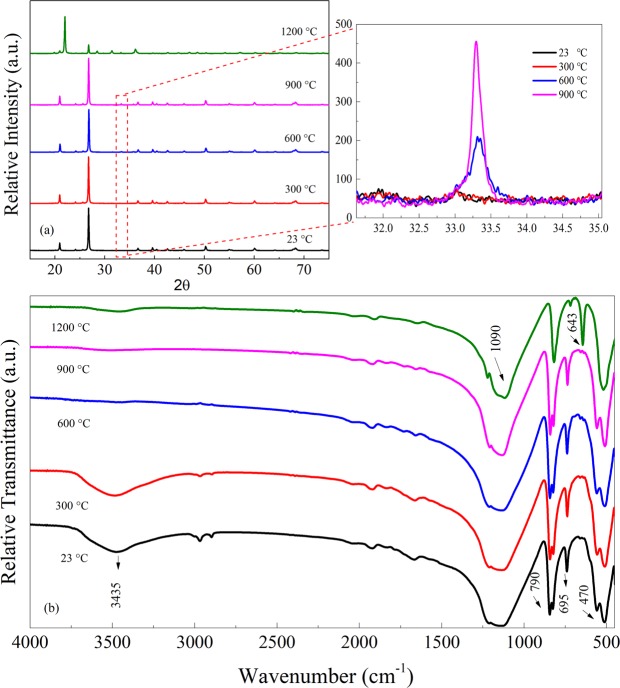
PXRD patterns (**a**), FTIR (**b**) spectra of mineral quartz heated to different temperatures. The enlarged part shows part of the plots in detail.

In order to characterize the water status of mineral quartz at different temperatures, PXRD results presented in Fig. 5a are used to analyze the crystalline degrees. Sharp diffraction characteristic peaks are exhibited in five plots, which indicate that the mineral quartz is highly crystalline. The positions and intensities of peaks have not altered significantly from 23 to 900 °C. Only a minor diverge is observed as shown in the inset. This suggests that mineral quartz has basically identical crystal form at those temperatures. With the temperature increasing from 600 up to 900 °C, the crystal gradually transforms from α-SiO_2_ to cristobalite as shown in the enlarged part. However, it is worth mentioning that a great variance occurs under 1200 °C. It indicates that a new substance “cristobalite” has been formed at such a high temperature^33^.

To directly confirm the water status in mineral quartz, FTIR results of the samples at different temperatures are shown in Fig. 5b. Three kinds of vibrations are characterized in it. Intense and wide bands of all samples at 1090 cm^−1^ are assigned to the asymmetrical stretching vibrations of Si-O-Si. The bands at 790, 695, 515, and 470 cm^−1^ are attributed to the symmetric stretching vibrations of Si-O bond^34^. Broad asymmetrical stretching vibration bands of -OH at 3435 cm^−1^ are noticed at 23 and 300 °C, which are attributed to the HFM of water molecules. The spectrum of 300 °C has a little difference with that of 23 °C. But the intensities of -OH become weaker than that of 23 °C. This is because the water content in the sample has been gradually reduced by raising temperatures. With the temperature increasing to 600 °C, the asymmetric stretching vibration bands of -OH at 3435 cm^−1^ have disappeared. It means that water has been removed completely at such an ambient temperature. The spectrum of 900 °C presents almost the same position of the bands as that of 600 °C. At 1200 °C, the bands of Si-O at 650 cm^−1^ start to shift to a lower wavenumber of 643 cm^−1^. And two split bands of Si-O have merged into one at around 470 cm^−1^. Based on those PXRD results, it is known that the mineral quartz has been converted into cristobalite under this case.

**Figure 6:**
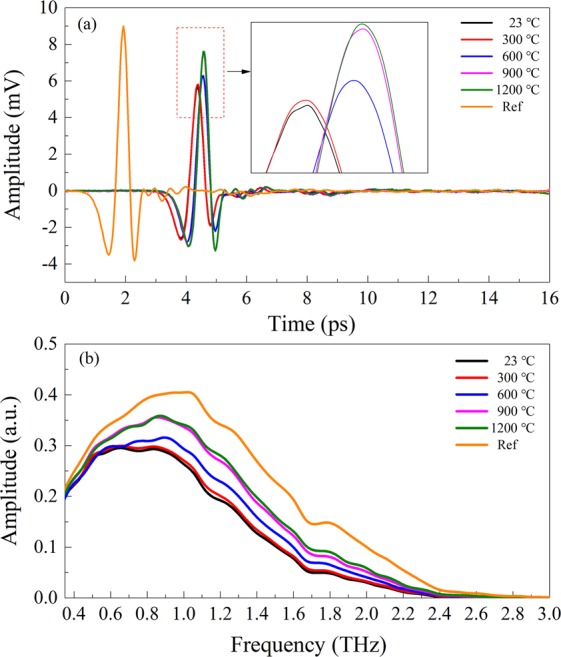
Diagram of THz-TDS (**a**) and THz-FDS (**b**) of mineral quartz heated to different temperatures. The inset shows the enlarged part of the plot detail.

The LFM of water and thermal formation within mineral quartz are characterized using THz-TDS as shown in Fig. 6a. It can be seen that although the phase delay occurs at 23 and 300 °C, the distortions of effective terahertz absorptions can be negligible. The tendency remains consistent with that of Fig. 2a. The higher the temperature, the higher the detected intensity of the temporal signal (inset in Fig. 6a). From the amplitudes especial of 300, 600 and 900 °C, it shows that the amplitudes relate with the temperature due to the variance of water content. This is in agreement with the FTIR spectra in Fig. 5b. It is also found that the amplitudes of THz-TDS become intense with the temperature increasing from 600 to 900 °C, even to 1200 °C. This can be attributed to the transformation of the structure of mineral quartz at such a high temperature. The similar amplitudes of 900 and 1200 °C suggest that water content no longer decreases and the structure of mineral quartz tends to stabilize till the cristobalite is formed under such conditions.

The amplitudes of THz-FDS reduce upon the increase of temperatures as shown in Fig. 6b. Compared to that of CuSO_4_·5H_2_O, the spectral limits of mineral quartz at five temperatures decay to just about 2.6 THz. The differences of the absorption between high and low temperatures are not obvious. This indicates that the relative steady structure of mineral quartz is not easily transformed with the increase of the temperature.

**Figure 7:**
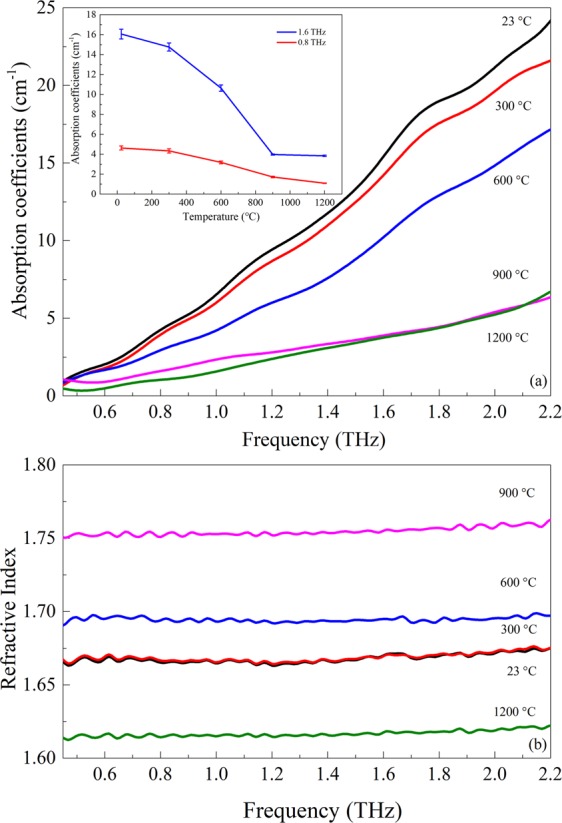
Frequency-dependent absorption coefficients (**a**) and refractive index (**b**) of mineral quartz heated to different temperatures. The inset shows the temperature-dependent absorption coefficients at 0.8 and 1.6 THz, respectively.

Figure 7 demonstrates the corresponding frequency-dependent absorption coefficients and refractive indices of mineral quartz. The absorption coefficients presented in Fig. 7a can be well interpreted by water content. In addition, the absorption coefficients of five temperatures show a nonlinear increasing over all frequencies. At lower frequencies below 1.6 THz, the absorption coefficients of 900 and 1200 °C increase slightly with the increasing frequency, which corresponds to the little drop in the absorption of the terahertz temporal signals (Fig. 6a). Notably, because the bending vibrations of the hydroxyl group of mineral quartz are more pronounced at higher frequencies^35,36^. The absorption coefficients are rather difficult to distinguish from 1.6 THz. As the decomposition of water has been realized completely by heating to 900 and 1200 °C, absorption coefficients tend to maintain invariable at higher frequencies. To clearly present the differences between high and low frequencies, the absorption coefficients at 0.8 THz and 1.6 THz are selected as shown in the inset. The absorption coefficients at 1.6 THz attenuate more than that of 0.8 THz, which is similar to the situation of Fig. 3a. The refractive indices in Fig. 7b show that mineral quartz of 1200 °C is more transparent to the terahertz radiation than that of other temperatures. Meanwhile, the similar refractive indices in the case of 23 and 300 °C illustrate the same status of mineral quartz during this heating process. The uniform trend of refractive indices suggests that mineral quartz at different temperatures does not seem to have specific absorption bands that could be attributed to any features.

**Figure 8:**
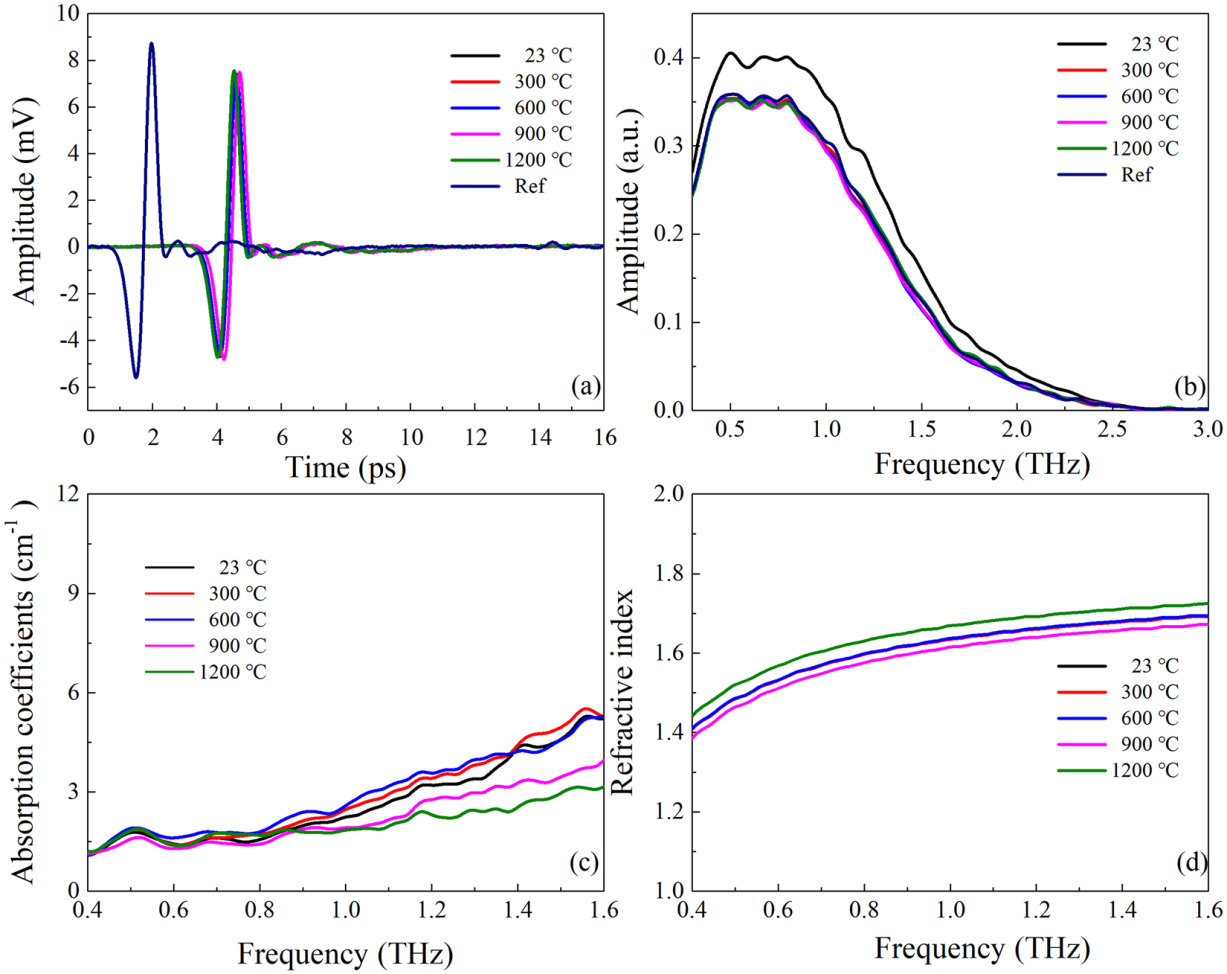
Terahertz time-domain spectra (**a**); frequency-domain spectra (**b**); absorption coefficients (**c**) and refractive index (**d**) of anhydrous mineral quartz after heating treatments (from 23 to 1200 °C) and then cooling to room temperature.

Similarly, as shown in Fig. 8, terahertz spectra present approximately same values for anhydrous mineral quartz at different temperatures. Consequently, the effect of dielectric properties of dehydrated mineral quartz could be neglected.

## Conclusions

In conclusion, based on THz-TDS, we have demonstrated the quantitative water determination of CuSO_4_·5H_2_O within the temperature range from 23 to 260 °C. Based on such results, water status incorporated in mineral quartz is characterized. The presented results show that in terms of hydrous minerals, the dehydration can be analyzed by the optical parameters of absorption coefficients and refractive index, even accompanying the structural transformation. This proposed study not only highlights the effectiveness of THz-TDS in clarifying the mechanism of the dehydration process of hydrous minerals, but also provides a potential in estimating the water reservoir of mineral system.

## Methods

### Sample preparations

CuSO_4_·5H_2_O crystals with high purity (≥99.99%) were used as raw materials in our experiments. Mineral quartz was the original mineral mined from Xinjiang area in China. Two kinds of minerals were crushed into powders by a grinder and the particle sizes approximate 75 μm were determined by a 200-mesh sieve to decrease the scattering absorption arising from the particles scattering to the terahertz wave. By comparing the literature data, when heating CuSO_4_·5H_2_O crystals, it will lose two water molecules at 60 °C firstly. And another two is at 100 °C, then the last one is at 260 °C^25^. In order to get trihydrate, monohydrate, and anhydrate, CuSO_4_·5H_2_O powders were heated to 60, 100 and 260 °C by the muffle furnace successively, with heating rate of 10 °C/min, and kept for 30 min to complete the phase transition. Similarly, heating treatments were applied to mineral quartz powders, but the temperatures of 300, 600, 900, and 1200 °C were selected. Subsequently, anhydrous copper sulfate and anhydrous mineral quartz were heated to corresponding temperatures as CuSO_4_·5H_2_O and mineral quartz powders. Finally, all the sample powders were cooled down to room temperature.

### THz-TDS

After the dehydration process, sample powders were compressed into about 1 mm thickness pellets with the diameter of 13 mm to be measured using THz-TDS. Each sample had a mass concentration of relevant powders of 0.2 g/(0.2 g + 0.1 g) ≈ 66.7%. The relevant powders (such as 0.2 g copper sulfate pentahydrate powders or mineral quartz powders) and 0.1 g polytetrafluoroethylene (PTFE) were uniformly mixed.

The THz-TDS experiments were performed with a transmission configuration at room temperature, and the spectrometer was continuously purged with nitrogen to minimize absorption from atmospheric water and keep the humidity less than 5% as depicted in Fig. 1. Initially, a mode-locked femtosecond Ti-sapphire laser with the central wavelength of 800 nm, 100 fs pulse duration and 80 MHz pulse repetition rate was used for the terahertz radiation generation and detection. Then the femtosecond laser was divided into two beams by the beam-splitter (BS), one of which was used as the pump beam with the higher power while the other as the probe beam with the lower power. The pump beam passing through the time-delay stage excited the photoconductive GaAs crystal to generate terahertz radiation. After being focalized and reflected by a set of parabolic mirrors (PM1-PM4), the collimated terahertz radiation transmitted the sample carrying sample information. Finally, the probe beam with the pump beam reached the ZnTe detecting crystal at the same time after transmitting through the silicon wafer. The thickness of the ZnTe detecting crystal is 2 mm and the orientation is <110>. The timing between the probe and the pump is given by a computer-controlled time-delay stage, allowing to sample the transient terahertz waveform in the timedomain. The reference (no sample put in the cell) and the sample signals were collected, respectively.

### PXRD

The pretreated powders of mineral quartz heated to 23, 300, 600, 900 and 1200 °C were respectively identified by the PXRD diffractometer (40 KV) with CuKα_1_ radiation (λ = 1.5406 Å) and CuKα_2_ radiation (λ = 1.5444 Å). The ambient temperature was 20 °C. The scanning range of the diffraction angles was 15–75°/2θ with the step size of 0.02°.

### FTIR

The mineral quartz powders after different heating temperatures were added KBr to make the samples. In the infrared spectrum measurement, the solid powders could not be directly used for tableting. It must be mixed with diluent (KBr powders) mainly due to the two reasons. On the one hand, the particle size of the powders is similar to the wavelength in the infrared range. Light scattering is severe. On the other hand, if the amount of sample is large, the phenomenon of total absorption of infrared light will occur affecting its SNR. Mixed samples were immediately recorded by Bruker TENSOR 27 infrared spectrometer (Germany) together with the same KBr sample as a reference. The measuring wavenumbers range from 500 to 4000 cm^−1^ with a resolution of 4 cm^−1^.

## Supplementary information

supplementary

## Data Availability

This work includes a data availability statement.
