# Supplementary material for: Insights into the water status in hydrous minerals using terahertz time-domain spectroscopy
Source: Sci Rep. 2019 Jun 25;9:9265. doi: 10.1038/s41598-019-45739-2 (PMC6592908; doi:10.1038/s41598-019-45739-2)
Supplement: Supplementary file 1 — supplementary [file 41598_2019_45739_MOESM1_ESM.docx]

**Insights into the water status in hydrous minerals using terahertz time-domain spectroscopy**

Yuanyuan Ma,^1^ Haochong Huang,^1,*^ Sibo Hao,^1^ Kunfeng Qiu,^2^ Hua Gao,^1^ Lu Gao,^1^ Weichong Tang,^1^ Zili Zhang,^1^ Zhiyuan Zheng,^1,**^





Figure 1. The crystallization water contents of CuSO_4_ are fitted to the absorption coefficients at different frequencies.

The relationship between the absorption coefficient and water content agreement with the Lambert Beer's law.





Figure 2. The differences in terahertz spectra between crystal quartz and mineral quartz.
